# Racial differences in healthcare expenditures for prevalent multimorbidity combinations in the USA: a cross-sectional study

**DOI:** 10.1186/s12916-023-03084-2

**Published:** 2023-10-23

**Authors:** Manal Alshakhs, Patricia J. Goedecke, James E. Bailey, Charisse Madlock-Brown

**Affiliations:** 1 Health Outcomes and Policy Program, University of Tennessee Health Science Center, Memphis, TN USA; 2 Department of Preventive Medicine, University of Tennessee Health Science Center, Memphis, TN USA; 3 Center for Health System Improvement, University of Tennessee Health Science Center, Memphis, TN USA; 4 Department of Diagnostic and Health Sciences, University of Tennessee Health Science Center, 66 North Pauline St. Rm 221, Memphis, TN 38163 USA

**Keywords:** Multimorbidity, Healthcare costs, Race, Ethnicity stratification, Age, Obesity status

## Abstract

**Background:**

We aimed to model total charges for the most prevalent multimorbidity combinations in the USA and assess model accuracy across Asian/Pacific Islander, African American, Biracial, Caucasian, Hispanic, and Native American populations.

**Methods:**

We used Cerner HealthFacts data from 2016 to 2017 to model the cost of previously identified prevalent multimorbidity combinations among 38 major diagnostic categories for cohorts stratified by age (45–64 and 65 +). Examples of prevalent multimorbidity combinations include lipedema with hypertension or hypertension with diabetes. We applied generalized linear models (GLM) with gamma distribution and log link function to total charges for all cohorts and assessed model accuracy using residual analysis. In addition to 38 major diagnostic categories, our adjusted model incorporated demographic, BMI, hospital, and census division information.

**Results:**

The mean ages were 55 (45–64 cohort, *N* = 333,094) and 75 (65 + cohort, *N* = 327,260), respectively. We found actual total charges to be highest for African Americans (means $78,544 [45–64], $176,274 [65 +]) and lowest for Hispanics (means $29,597 [45–64], $66,911 [65 +]). African American race was strongly predictive of higher costs (*p* < 0.05 [45–64]; *p* < 0.05 [65 +]). Each total charge model had a good fit. With African American as the index race, only Asian/Pacific Islander and Biracial were non-significant in the 45–64 cohort and Biracial in the 65 + cohort. Mean residuals were lowest for Hispanics in both cohorts, highest in African Americans for the 45–64 cohort, and highest in Caucasians for the 65 + cohort. Model accuracy varied substantially by race when multimorbidity grouping was considered. For example, costs were markedly overestimated for 65 + Caucasians with multimorbidity combinations that included heart disease (e.g., hypertension + heart disease and lipidemia + hypertension + heart disease). Additionally, model residuals varied by age/obesity status. For instance, model estimates for Hispanic patients were highly underestimated for most multimorbidity combinations in the 65 + with obesity cohort compared with other age/obesity status groupings.

**Conclusions:**

Our finding demonstrates the need for more robust models to ensure the healthcare system can better serve all populations. Future cost modeling efforts will likely benefit from factoring in multimorbidity type stratified by race/ethnicity and age/obesity status.

**Supplementary Information:**

The online version contains supplementary material available at 10.1186/s12916-023-03084-2.

## Background

The scarcity of economic models in multimorbidity research represents a serious challenge [1]. A significant limitation of current models is that most do not consider the varying costs of different disease combinations [2]. A better understanding of the burden of multimorbidity through cost assessment for various multimorbidity combinations will assist in targeting highest cost patients for intensive interventions [1]. Most high healthcare utilizers have at least two chronic conditions [3]. Among US adults, multimorbidity is estimated to have a prevalence of 58.4% [4]. Addressing the economic burden of multimorbidity is crucial to developing effective strategies for managing care.

Factoring in multimorbidity has been shown to explain these expenditures better than models based on population characteristics (size and demographics) alone [5]. For example, in the case of diabetes, different comorbidities have a varying impact on cost [6]. Previous studies evaluating the impact of specific multimorbidity combinations on expenditures thus far have focused on a few diseases [2]. The most prevalent multimorbidities in the USA represent a broad spectrum of diseases [7]. Effective care planning and resource management requires accurately projecting patient costs for these disease combinations [2, 8].

Modeling the associations of most prevalent multimorbidity combinations with healthcare expenditures is essential to further aging research because the majority of the 65 + population have two or more chronic conditions and account for 47% of Medicare spending [9]. Interventions aimed at slowing the aging process need to target patients with multiple diseases to be effective [10]. Mercer et al. (2016) found that multimorbidity-focused interventions are cost-effective for this patient population [11].

Better modeling of expenditures is essential for improving the health of racial and ethnic minorities. Clay et al. found that among African American men, comorbidity clusters are associated with poor outcomes, including poor health-related quality of life, disability, and higher mortality rate. As these authors suggest, better modeling of expenditures will be essential for improving the health of racial and ethnic minorities [12]. Multimorbidities exacerbate health inequalities as underserved populations are at greater risk for multimorbidity, increasing their disease burden [13]. Despite a clear need to better understand health disparities, research shows that even robust methods can be susceptible to bias. Predictive models derived from primarily homogenous populations may be poorly generalizable and can exacerbate racial/ethnic disparities [14]. Cost estimates of multimorbidity must address model racial/ethnic bias. To date, no large-scale study of the expenditures associated with common multimorbidity combinations has assessed the accuracy of model predictions across races and obesity status.

This research compares total healthcare expenditures for the most prevalent multimorbidity combinations across racial/ethnic groups. We also aim to determine if multimorbidity expenditure models have similar accuracy across racial/ethnic groups after adjustment for potential confounding factors. In addition, the study aims to assess for possible differences in total charges for 45–64 versus 65 + patients as the incidence of chronic disease rises exponentially with age [15]. This study is among the first to model total charges associated with the most prevalent multimorbidity combinations by race/ethnicity. Our previous work identified the most prevalent multimorbidity combinations by race/ethnicity, serving as the foundation for this current research [7]. Our primary objectives are to identify the expected total charges associated with the most prevalent multimorbidity combinations by race/ethnicity. Additionally, we sought to assess differences in expenditures for these multimorbidity combinations and assess differences in model accuracy by race/ethnicity.

## Methods

### Research design

This cross-sectional study employed de-identified data for 2016–2017 from the Cerner HealthFacts® data warehouse. The dataset includes electronic health records (EHR) representing over 490 million patient encounters for over 70 million patients treated at hospitals and clinics at 792 non-affiliated healthcare systems throughout the USA. Variable categories include encounter type, medical history, diagnoses, labs, prescriptions, patient demographics, clinic type, and procedures. Inclusion criteria for patients included the following: (1) age 45 + , (2) body mass index (BMI) value present and between 18.5 and 206, (3) EHR-identified race category, (4) EHR-identified gender, (5) patient encounters not missing total charges, and (6) an encounter with an International Classification of Diseases-10th Version-Clinical Modification (ICD-10–CM) diagnosis code among the 38 broad diagnoses that make up the most prevalent multimorbidities in the USA. These diagnoses involved in prevalent multimorbidities were identified in our previous research and are listed in Additional file 1: Table S1 [7]. In our previous research, we identified disease combinations, frequent above the threshold of 5%, shared by all races/ethnicities for each age/obesity level. The current work considers the economic impact of those multimorbidity combinations in their respective age/obesity level cohort. We aggregated ICD-10-CM sub-classifications of diseases into broad categories for all 38 diagnoses; for example, I11.9 (hypertensive heart disease without heart failure) fell under the broader parental category I11 (hypertensive heart disease). Using a prevalence-based approach for assessing multimorbidity validated through prior research, [16–18] we defined multimorbidity as the presence of two or more ICD-10–CM diagnosis codes in an individual during the 2-year (i.e., 2016–2017) study period. Our upper BMI cutoff is based on the highest recorded BMI value was between 206 and 224, so we considered it valid if the BMI value was 206 or less [19, 20]. Since our interest was addressing multimorbidities associated with obesity, underweight patients were excluded (BMI < 18.5) as they might have different multimorbidity issues. The 2-year assessment period was employed to maximize the probability of identifying all major prevalent multimorbidities experienced by individuals during the study period. Since diseases might not be diagnosed at the same visit or within the same year, this longer period allowed us to capture more data than would a single year.

### Ethical considerations

The data were de-identified and excluded the 16 identifiable variables that necessitate Internal Review Board (IRB) approval for access. Because the study only employed de-identified data, the study was considered not human subjects’ research. Per the National Institutes of Health Office of Human Subjects Research policy, the University of Tennessee Health Science Center (UTHSC) Institutional Review Board (IRB) determined that the research was exempt. We performed this research following all other relevant research requirements.

### Independent variables

Demographic, multimorbidity, and healthcare utilization variables were the primary independent variables. Demographic variables included race, age, gender, BMI, payer information, and rural or urban status. BMI was treated as a dichotomous variable, classifying patients with obesity (30 ≤ BMI < 206) and without obesity (18.5 ≤ BMI < 30). When assessing the financial burden across races in adults aged (45–64) and 65 + , controlling for factors impacting disease severity and socioeconomic issues affecting cost is critical [21]. Therefore, we assessed payer status, rurality, length of stay, and the Elixhauser Comorbidity Index (ECI) score. Hospital information and healthcare usage variables included the number of inpatient and outpatient visits, emergency visits, teaching hospital status, care-type status, and total hospital admission days, if any. Because ethnicity is not a separate variable in the Cerner HealthFacts database, Hispanic is listed as a racial category. Other racial categories included Caucasian, African American, Biracial, Asian/Pacific Islander, and Native American. Patients were stratified into two cohorts [age 45–64] and [age 65 +]) according to their age at the beginning of the study. Only patients with an EHR-identified gender (i.e., male and female) were included in the study. Additional file 1: Table S2 clarifies the remaining variables.

### Dependent variable

Our primary dependent variable was the sum of total charges for all encounters over the 2-year study period for each patient. Healthcare utilization information included the total charges for each encounter. We categorized patient encounters into one of three categories: inpatient, outpatient, or emergency visit. We chose our dependent variable to be total charges since it is the amount that reflects the expense of the service received before any discounts or negotiations. Arora et al. (2015) described the challenge of answering the question “how much does healthcare cost?” and divided healthcare expenditures into three categories: price or charge, cost, and reimbursement [22]. Price or charge is defined as the amount billed by a provider for a healthcare service. Hospitals in the USA use a price list called chargemaster that includes a list of all billable services before any discounts or negotiation to arrive at the price charged, which varies across hospitals [23, 24]. The definition of cost varies with perspective. For the provider, the cost is simply the expense incurred to deliver healthcare services to the patient; for the payer, it is the amount that they will pay providers for these services; and for the patient, it is the amount they pay out-of-pocket for healthcare services rendered. Finally, reimbursement is defined as the amount paid a provider by a third party (the payer) for the services rendered to the patient. Due to different agreements and negotiations between hospital providers and payers, cost and reimbursement can vary across patients receiving the same service from the same hospital [22, 25].

### Missing data

Due to their minimal numbers, we deleted hospitals with no census division or rural/urban status information. We imputed hospitals with teaching facility information missing by adding the most prevalent category [26]. We excluded encounters with $0 listed for total charges from the study. According to the Cerner HealthFacts® database data dictionary, total charges of $0 indicate that the administrative staff did not enter the billing information into the database. We compared demographics for sources with missing cost data and those with cost data present; the demographics were not statistically different. For ease of interpretation, the patient record was removed from the study if a patient was treated in two different census divisions or if the patient was treated in a rural and an urban hospital.

### Statistical analysis

We examined the distribution of our dependent variable, total charges over the 2 years. We checked for skewness and outliers. Having so many variables, we also tested for multicollinearity, a linear relationship between two or more variables [27]. We used a generalized variance inflation factor (GVIF) analysis to identify variables with high multicollinearity, which is appropriate for a mix of categorical and numerical variables [28, 29]. We removed the variable with the highest GVIF^(1/2Df) score using the car R package [30]. We repeated this process until no variable had a score above two, a conservative threshold for considering multicollinearity [29].

We used regression analysis to compare the total charges of the most prevalent multimorbidity combinations by race/ethnicity. A generalized linear model (GLM) with gamma distribution and log link function was applied to estimate the total charges based on the morbidity variables [31, 32]. ECI rank was categorized into three categories based on quantile range: low, medium, and high, indicating comorbidity severity. We ran a 3-way ANOVA test on the model residuals to determine whether there was an interaction effect between BMI and race, as a combined effect, and ECI ranks on total charges (the dependent variable).

## Results

### Demographics

In this study, most patients in both age cohorts were female. Tables 1 and 2 show the breakdown of demographics by race for the 45–64 and 65 + cohorts, respectively. The percentages were calculated relative to the whole patient population. The average age for the 45–64 cohort (333,094 patients) was 55 years and for the 65 + cohort (327,260 patients) was 75 years.

**Table 1:** Demographics of the 45–64 cohort

Race	Prevalence *n* (%)	Gender		Payer info			Area status	
		**Female** ***n* (%)**	**Male** ***n* (%)**	**Medicaid/Medicare/Title V** ***n* (%)**	**Other** ***n* (%)**	**Unknown** ***n* (%)**	**Urban** ***n* (%)**	**Rural** ***n* (%)**
African American	44,595 (13)	26,260 (8)	18,335 (6)	12,145 (4)	28,597 (9)	3853 (1)	40,203 (12)	4392 (1)
Asian/Pacific Islander	3976 (1)	2341 (1)	1635 (< 1)	504 (< 1)	2864(1)	608(< 1)	2,925 (1)	1051 (< 1)
Biracial	346 (< 1)	189 (< 1)	157 (< 1)	43 (< 1)	257 (< 1)	46 (< 1)	265 (< 1)	81 (< 1)
Caucasian	278,676 (84)	150,101 (45)	128,575 (39)	55,010 (17)	209,433 (63)	14,233 (4)	227,618 (68)	51,058 (15)
Hispanic	485 (< 1)	274 (< 1)	211 (< 1)	78 (< 1)	369 (< 1)	38 (< 1)	422 (< 1)	63 (< 1)
Native American	5016 (2)	2784 (1)	2232 (1)	1393 (< 1)	3524 (1)	99 (< 1)	2735 (1)	2281 (1)
**Total**	333,094 (100)	181,949 (55)	151,145 (45)	69,173 (21)	245,044 (74)	18,877 (6)	274,168 (82)	58,926 (18)

**Table 2:** Demographics of the 65 + cohort

Race	Prevalence *n (*%)	Gender		Payer info			Area status	
		**Female** ***n* (%)**	**Male** ***n* (%)**	**Medicaid/Medicare/Title V** ***n* (%)**	**Other** ***n* (%)**	**Unknown** ***n* (%)**	**Urban** ***n* (%)**	**Rural** ***n* (%)**
African American	23,529 (7)	14,381 (4)	9148 (3)	17,655 (5)	4004 (1)	1870 (1)	21,174 (6)	2355 (1)
Asian/Pacific Islander	3729 (1)	2309 (1)	1420 (< 1)	2426 (1)	635 (< 1)	668 (< 1)	2277 (1)	1452 (< 1)
Biracial	1601 (< 1)	84 (< 1)	76 (< 1)	54 (< 1)	61 (< 1)	45 (< 1)	94 (< 1)	66 (< 1)
Caucasian	297,299 (91)	164,042 (50)	133,257 (41)	241,109 (74)	47,966 (15)	8224 (3)	243,273 (74)	54,026 (17)
Hispanic	220 (< 1)	111 (< 1)	109 (< 1)	141 (< 1)	68 (< 1)	11 (< 1)	187 (< 1)	33 (< 1)
Native American	2323 (1)	1352 (< 1)	971 (< 1)	1779 (1)	513 (< 1)	31 (< 1)	1265 (< 1)	1058 (< 1)
**Total**	327,260 (100)	182,279 (55)	144,981 (45)	263,164 (81)	53,247 (16)	10,849 (3)	268,270 (81)	58,990 (19)

### Outcomes

The breakdown of visit type, mean emergency room visits, mean ECI score, mean admission days, and mean charges for the 45–64 and 65 + cohorts are shown in Table 3. The Cerner HealthFacts® database included data from 1,500,580 45–64 patients and 1,213,069 65 + patients for the period 2016–2017. We excluded some of the ICD-9-CM diagnosis codes and removed patient encounters with missing total charges for a total of 647,801 patients remaining in the 45–64 cohort and 534,534 patients remaining in the 65 + cohort. Patients excluded due to not having a morbidity, BMI, race, gender, or age value totaled 189,213 in the 45–64 cohort and 98,222 in the 65 + cohort. In the 45–64 cohort, 68,856 patients were excluded due to duplicate hospital information, and 60,388 in the 65 + cohort. After excluding patients based on this inclusion/exclusion criteria, 333,094 patients were 45–64, and 327,260 remained. A complete breakdown of our exclusion/inclusion criteria on the patient population is displayed in Additional file 1: Fig. S1.

**Table 3:** Outcomes of the 45–64 and 65 + cohorts

Race	The 45–64 cohort				The 65 + cohort			
	**Mean E.R. visits**	**Mean ECI score**	**Mean hospital admission days**	**Mean charges**	**Mean E.R. visits**	**Mean ECI score**	**Mean hospital admission days**	**Mean** **charges**
African American	1	2	1	$78,544	1	6	2	$176,274
Asian/Pacific Islander	1	2	0	$54,410	1	4	1	$167,949
Biracial	0	2	0	$47,238	1	4	1	$140,628
Caucasian	1	1	0	$55,704	1	4	1	$146,224
Hispanic	0	1	0	$29,597	0	3	1	$66,911
Native American	1	2	0	$50,496	1	4	1	$111,522

Due to the skewness of the dependent variable (mean total charges), we performed an outlier test and used the interquartile method to eliminate outliers. After testing for collinearity, we removed the *total number of morbidities* variable from the analysis, as it was considered an aliased coefficient in the model, meaning that this particular variable was equivalent to one or more variable(s). We determined the unadjusted and adjusted models’ residuals for the 45–64 and 65 + cohorts using a generalized linear model with Gamma distribution and log link function (Additional file 1: Fig. S1) and assessed model performance by inspecting the residuals’ quantile–quantile (Q-Q) plots in R. Due to the skewness of the dependent variable, total charges, these models did not fit the data well. To obtain a better-fitting model, we tested log and exponential transformations. We then inspected the residuals’ Q-Q plots to measure the model performance (Additional file 1: Fig. S2 and Additional file 1: Fig. S3). We selected the exponential model as optimal for this dataset, since it exhibited the least sum of square error (SSE) in both cohorts [33].

### The model

The healthcare total charges model in the 45–64 and 65 + cohorts had adjusted *R*-squared values of 0.3906 and 0.4695, respectively. Tables 4 and 5 show the model estimates for key demographic and patient hospital utilization factors. African American was selected as the index race. The Asian/Pacific Islander and Biracial variables (Table 4) were not significant in the 45–64 cohort. The Caucasian, Hispanic, and Native American races had negative total charges estimates. In the 65 + cohort (Table 5), the Biracial variable was not significant. The Asian/Pacific Islander and the Caucasian races had positive total charges estimates, while the Hispanic and the Native American races had negative estimates. Additional file 1: Tables S3 and S4 include model estimates for hospital-related variables and the 38 diagnoses that comprise the most prevalent multimorbidities across race/ethnicity in the USA. For each model, all diagnosis estimates were significant. All diagnosis estimates for the 45–64 cohort (Additional file 1: Table S3) were positive except heart failure, vitamin D deficiency, and chronic kidney disease, which were all slightly negative. Hospital teaching status was not a significant predictor. For the 65 + cohort (Additional file 1: Table S4), all diagnoses were positive except vitamin D deficiency and chronic kidney disease. Living in the South Atlantic region was not a significant predictor for healthcare charges for this cohort.

**Table 4:** The 45–64 cohort total charge model estimates

Demographics (variable)	Estimate	*P*-value	Significance
Age	− 0.001	< 0.001	***
Race/Asian Pacific Islander	0.006	0.188	-
Race/Caucasian	− 0.006	< 0.001	***
Race/Native American	− 0.015	< 0.001	***
Race/Biracial	− 0.018	0.175	-
Race/Hispanic	− 0.154	< 0.001	***
Male	− 0.010	< 0.001	***
BMI	0.000	< 0.001	***
Length of stay	0.014	< 0.001	***
Payer/unknown	0.035	< 0.001	***
Payer/other	− 0.007	< 0.001	***
ECI	0.008	< 0.001	***
Emergency visits	0.028	< 0.001	***
Outpatient visits	0.003	< 0.001	***

^***^Indicates *p*-value < 0.05

**Table 5:** The 65 + cohort total charge model estimates

Demographics (variable)	Estimate	*P*-value	Significance
Age	0.001	< 0.001	***
Race/Asian Pacific Islander	0.123	< 0.001	***
Race/Caucasian	0.015	< 0.001	***
Race/Biracial	− 0.028	0.550	-
Race/Native American	− 0.046	0.001	***
Race/Hispanic	− 0.369	< 0.001	***
Male	− 0.007	0.005	**
BMI	− 0.001	< 0.001	***
Length of stay	0.025	< 0.001	***
Payer/unknown	0.042	< 0.001	***
Payer/other	− 0.075	< 0.001	***
ECI	0.018	< 0.001	***
Emergency visits	0.024	< 0.001	***
Outpatient visits	− 0.008	< 0.001	***

^***^Indicates *p*-value < 0.05

The overall mean of the absolute value of the model residuals and the standard deviations for the 45–64 and 65 + cohorts are shown in Table 6. The model best predicted the total charges for the Hispanic race and was least accurate for the African American race. This table also displays extreme standard deviation values for the model’s residuals. All standard deviations were greater than the mean, and some races exhibited remarkably high standard deviations.

**Table 6:** Mean model residuals for the 45–64 and 65 + cohorts

Race	45–64 cohort		65 + cohort	
	**Mean residuals**	**Standard deviation**	**Mean residuals**	**Standard deviation**
**African American**	175,514	19,636,829	157,118	646,311
**Asian/Pacific Islander**	45,864	108,516	120,527	271,469
**Biracial**	40,425	77,686	109,464	180,946
**Caucasian**	47,112	515,279	205,310	20,528,300
**Hispanic**	27,569	65,028	56,234	134,076
**Native American**	70,938	1,673,968	83,095	161,816

**Fig. 1:**
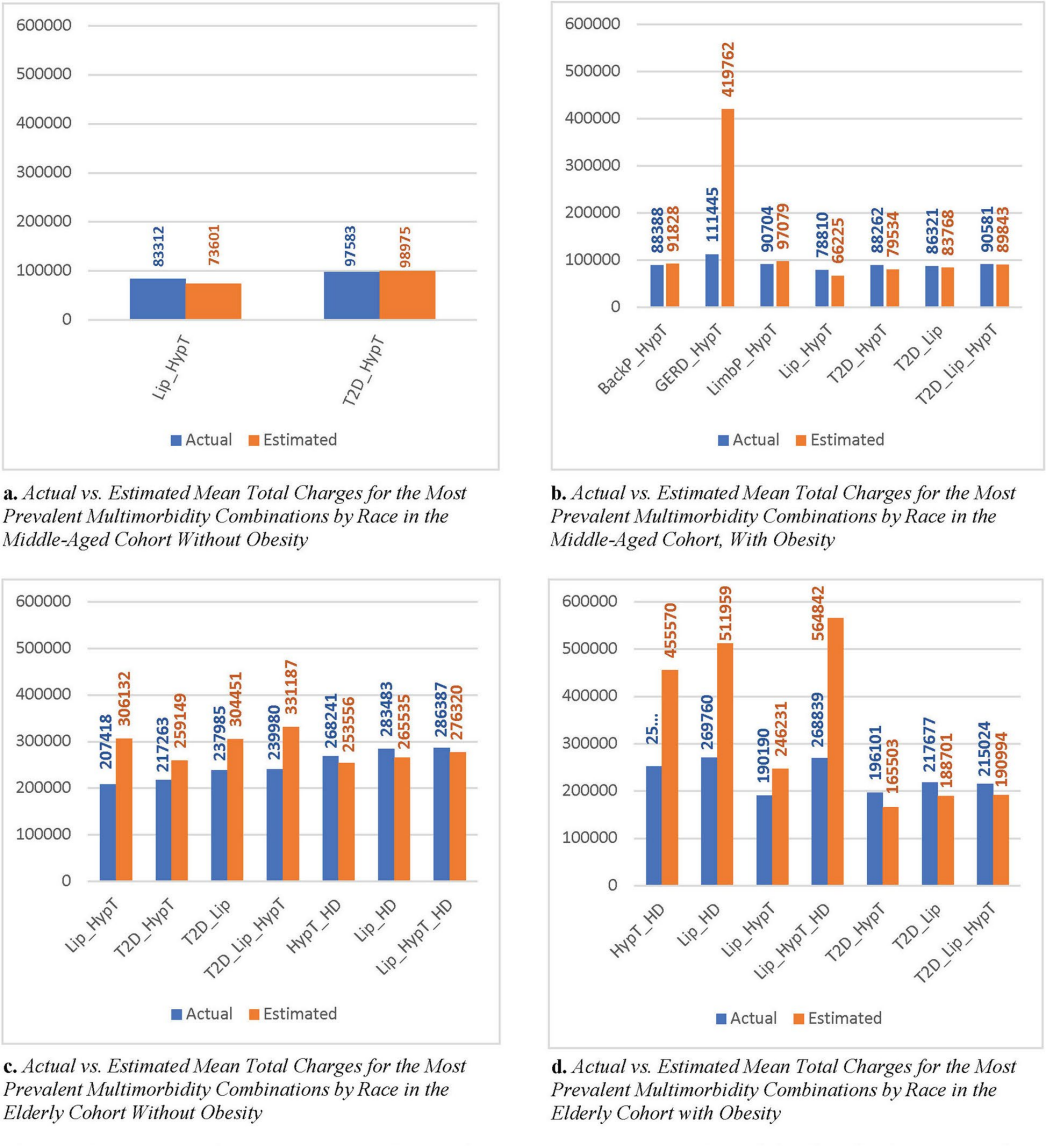
Actual and estimated mean total charges for the most prevalent multimorbidity combinations by race in the 45–64 and 65 + cohorts, with and without obesity. Abbreviations: BackP, severe back pain; GERD, gastroesophageal reflux disease; HD, heart disease; HypT, hypertension; Lip, lipidemia; LimbP, pain in limb, hand, foot, fingers, and toes; T2D, type 2 diabetes mellitus

The actual vs. the estimated mean total charges over the study period (2016–2017) for the most prevalent multimorbidity combinations shared by all races in the 45–64 and 65 + cohorts, respectively, are shown in Fig. 1. The multimorbidity combinations varied by age and obesity status as they represent the multimorbidities frequent at or above 5% for all racial/ethnic groups within each cohort based on our previous work. The variance between the actual and estimated mean total charges for the *hypertension* + *GERD* multimorbidity combination in the 45–64 cohort with obesity was almost double. In the 65 + cohort with obesity, *hypertension* + *heart disease*,* lipidemia* + *hypertension* + *heart disease*, and *lipidemia* + *heart disease* multimorbidities exhibited the highest variance between the actual and estimated mean total charges with values that were also almost double. In general, the mean total charges and the variance between actual and estimated mean total charges were higher in the 65 + cohorts than in the 45–64 cohorts.

**Fig. 2:**
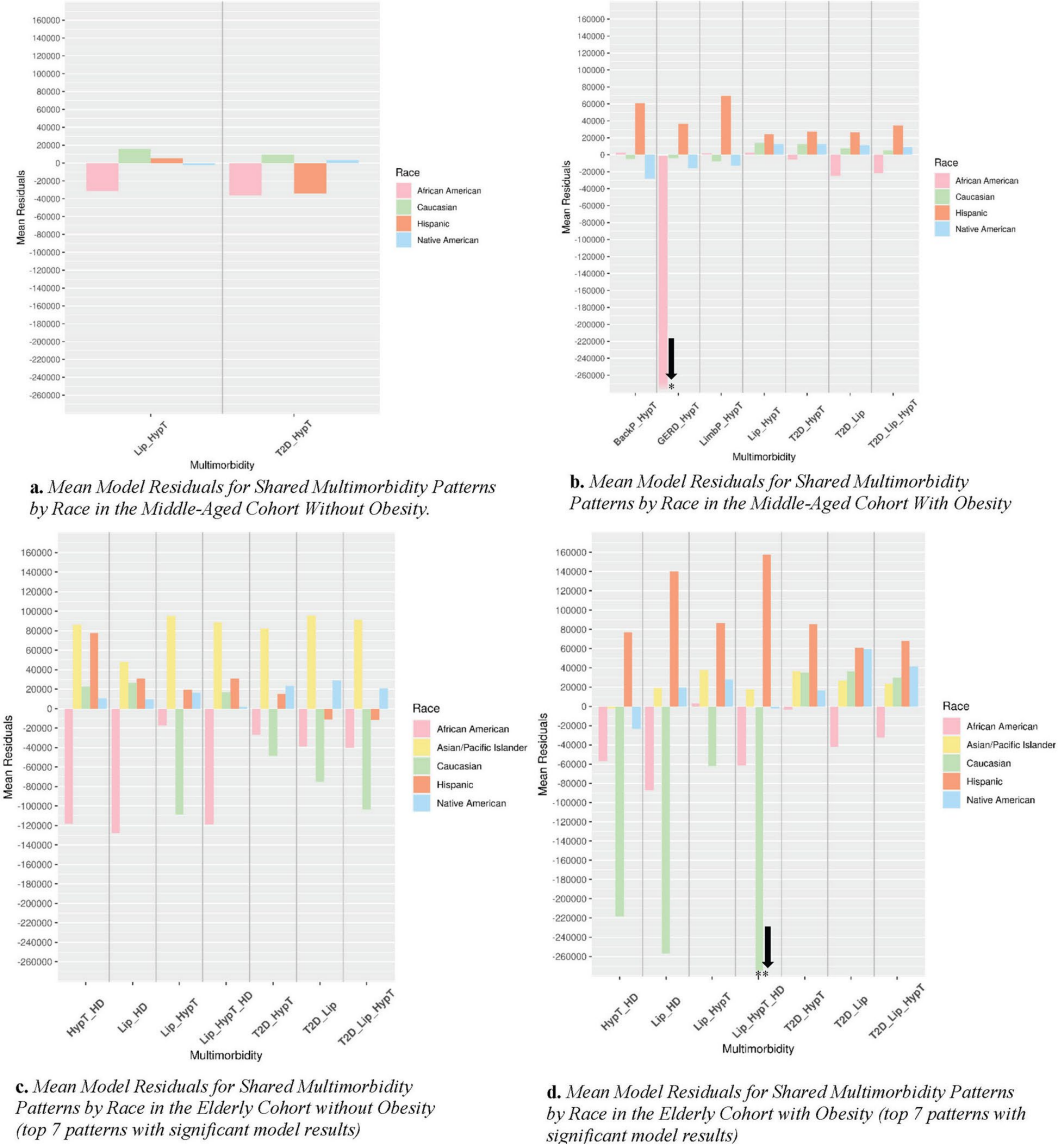
Mean model residuals for shared multimorbidity patterns by race in the 65 + and 45–64 cohorts with and without obesity. * =  − 1,749,059 residual value, ** =  − 316,995 residual value (these values were too large to display in the figure). Abbreviations: BackP, severe back pain; CKD, chronic kidney disease; GERD, gastroesophageal reflux disease; HD, heart disease; HypT, hypertension; Lip, lipidemia; LimbP, pain in limb, hand, foot, fingers, and toes; OJD, other joint disorder; T2D, type 2 diabetes mellitus

The mean residuals by race for each of the most prevalent multimorbidity combinations in both cohorts with and without obesity are shown in Fig. 2. Due to many prevalent multimorbidity combinations in the 65 + cohorts, we examined the residuals by race for only the top seven most prevalent. The mean model residuals for the shared multimorbidity patterns by race in the 45–64 cohort without obesity are shown in Fig. 2a. The model overestimated the total charges for both shared multimorbidities for the African American race and one shared multimorbidity for the Hispanic race, and it underestimated the total charges for the Caucasian race. The best estimates were for the Native American race. In contrast, the mean model residuals for the shared multimorbidity patterns by race in the 45–64 cohort with obesity (Fig. 2b) indicated that the model highly overestimated the total charges for the GERD + hypertension multimorbidity pattern in the African American race. The model underestimated all of the total charges for the multimorbidity patterns for the Hispanic race, while the estimates for the Native American race fluctuated between over- and underestimation.

The mean model residuals for the shared multimorbidity patterns by race in the 65 + cohort without obesity are shown in Fig. 2c. The total charge estimates for the African American race were overestimated or highly overestimated, while those for almost all of the Asian/Pacific Islander race were highly underestimated. The model also overestimated two patterns for the Hispanic race and highly underestimated the remainder. In contrast, the mean model residuals for the shared multimorbidity patterns by race in the 65 + cohort with obesity (Fig. 2d) indicated that the model underestimated all of the total charge estimates for the Asian/Pacific Islander race. The Hispanic race exhibited the most highly underestimated patterns, while those for the Caucasian race had one triad pattern that was significantly overestimated (− 316,995). The model estimated two multimorbidity patterns for the African American race better than others but overestimated the remaining patterns. The remaining patterns were either highly overestimated or underestimated.

The model estimated the mean total charges for the hypertension + GERD multimorbidity pattern as much higher than the actual charges. The mean model residual for the same cohort was highly overestimated for the African American race. The model also estimated the mean total charges for the lipidemia + hypertension + heart disease triad multimorbidity pattern as much higher than the actual charges. The mean model residual for the same cohort was highly overestimated for the Caucasian race. The multimorbidities with heart disease also showed extreme differences in actual vs. estimated mean total charges and an extreme over or underestimation for the mean model residuals of certain races.

The variability of mean model residuals for the African American race increased with obesity in the 45–64 cohort, yet variability decreased with obesity in the 65 + cohort. The model best estimated the lipidemia + hypertension multimorbidity pattern for this race across all cohorts. The Asian/Pacific Islander race model residuals were more extreme for patients in the 65 + cohort without obesity than those with obesity. Although the Asian/Pacific Islander race exhibited negative model estimates compared to the African American race, when the mean model residuals were categorized as a function of multimorbidity, some combinations were better estimated than others. For the Caucasian race, the variability of mean model residuals was comparable by weight class in the 45–64 cohort, but it was more accurate for patients without obesity in the 65 + cohort. The most accurate overall model estimates were for the Native American race, although variability in mean model residuals increased with obesity and aging. The mean model residuals also increased substantially by age group for the African American, Hispanic, and Native American races.

We conducted a post hoc sensitivity analysis to determine if some groups’ small numbers caused the extreme average residual values. We tested this hypothesis by re-running our models with the smaller groups (Native American and Hispanic) combined. Additional file 1: Fig. S4 displays the results. The extreme values remain extreme, and two additional multimorbidities (HypT_HD and Lip_HD) had extreme values for the Caucasian group in the 65 + with obesity cohort.

Since BMI and race as a combined effect were not significant (*p*-value = 0.870) when analyzed by 3-way ANOVA, we removed this interaction so that only ECI rank was significant (*p*-value = 0.0353) for the 45–64 cohort. For the 65 + cohort, BMI and race as a combined effect were not significant (*p*-value = 1.000,) and only ECI rank was significant (*p*-value < 0.001).

## Discussion

This study adds significantly to previous literature by elucidating the complex relationships between multimorbidity and costs across racial groups [34, 35]. The well-fit cost models developed through this study show that the accuracy of estimating cost varies across race and by multimorbidity, age group, and obesity status. However, it exhibited varying patterns of over- or underestimating total charges for specific racial groups. This suggests that more robust methods will be necessary to ensure accurate cost capture, particularly for vulnerable populations. Capturing such a complex interplay is challenging. While this type of modeling has some limitations, it can help to identify the costs associated with multimorbidities to help project future patient costs. This study also showed that aging does not have a straightforward relationship with cost estimates for certain races. For example, African Americans were the index race in both models. The Caucasian race had a negative total charges estimate for the 45–64 cohort and a positive total charges estimate for the 65 + cohort.

While previous literature notes that levels and most prevalent categories of multimorbidity vary by race [36], our research shows that the relationship between cost and multimorbidity is inconsistent for each racial group. Specific total charge estimates for some multimorbidity patterns were more inaccurate for some groups. Additionally, our study demonstrated that some racial groups could be driving the overall inaccuracy of cost estimates for specific multimorbidity combinations. For example, the average estimated total charges for hypertension + GERD significantly deviated from the actual total charges. Residual analysis indicated that these estimates were significantly overestimated for the African American population in particular. As multimorbidity is associated with higher outpatient and inpatient utilization of healthcare services, [37] the importance of accurately modeling cost cannot be overstated. Given the seriousness of the inequalities in healthcare access and outcomes by race, [38] it is crucial that we generate accurate models across racial groups. Our findings provide necessary information on understanding the complexity of the relationship between cost and multimorbidity. Researchers modeling multimorbidity and cost must analyze estimates for specific patterns stratified by race to know how much specific estimates can be trusted.

Our results indicate that the pattern of model accuracy across the obesity category varies by race. These findings can help the research community identify areas for improved modeling to better estimate costs for patient populations. Except for one multimorbidity combination (hypertension + GERD), multimorbidities in the patient population with obesity exhibited less extreme average residuals in the African American group in both the 45–64 and 65 + cohorts. We observed a similar relationship in the 65 + Asian population. On the other hand, the Hispanic population exhibited more extreme residuals in the absence of obesity. We observed a similar trend in the 65 + Native American and Caucasian populations. In some instances, this could be attributed to differences in the type of multimorbidities in distinct groups, but we also observed this trend in cases where the multimorbidity is the same (e.g., lipidemia + heart disease in the 65 + cohort). Our results demonstrate the importance of stratification by weight category for improved model accuracy.

### Limitations

The cross-sectional design of our study restricts our comprehension of multimorbidity, race, age, BMI, and ECI as risk factors that impacting patients’ mean total charges. The results could not produce a single model consistent in predicting total charges across races in the same weight and age groups. The Cerner HealthFacts database contains patient records with missing charges due to information not being transferred to the data warehouse. Consequently, we excluded these records from our study. However, as noted in the “Methods” section, this data is likely missing at random. A non-trivial percent of patients had missing data in the following categories: BMI, race, gender, or age. These variables are likely to have some missing not at random data which may bias our estimates. Some of this data could be missing at random as, for example, not all HealthFacts sites provide BMI values. The Uniform Hospital Discharge Data Set (UHDDS) definitions and regulations drive hospital reporting requirements for race and ethnicity data, which may not accurately reflect these categories [39]. The Cerner HealthFacts database categorized the Hispanic ethnicity as a race, yet these patients could identify as a member of the Native American, Black, White, or Asian races or could be Biracial. Our exclusion of patients with unknown race, gender, BMI, or age data substantially reduced our sample, which could impact specific groups disproportionally. For the 45–64 cohort’s model, the Asian/Pacific Islander and Biracial races were insignificant, nor was the Biracial racial category significant for the 65 + cohort’s model, which is most likely because we had small samples for these two races. Healthcare expenditures at the end of life can differ significantly from the cost of general medical care. This study was not able to include these expenditures since we do not have out-of-hospital death information.

Despite these limitations, our study is unique because it included the Biracial and the Native American groups, which are often not studied. Also, the study population reflects a nationwide sample selected from all corners of the nation and is representative of the patient group that doctors generally treat in a clinical setting.

If building a model for the most prevalent multimorbidity combinations by race is so challenging, how accurate will expenditure models be for multimorbidities that are not shared by all racial groups and how can we evaluate them? Although the model we developed was a good fit for the data we accessed, its variability in predicting total charges by race demonstrated that we need more robust models that accurately predict total healthcare charges for all racial groups. In particular, multimorbidity and race need to be studied more comprehensively in this regard.

## Conclusions

This is the first study to identify total charges’ trends across Asian/Pacific Islander, African American, Biracial, Caucasian, Hispanic, and Native American populations for the most prevalent multimorbidity combinations. We also demonstrated that our model was inconsistent in its ability to predict total charges by race based on multimorbidity patterns. In general, the total charges were either over- or underestimated across multimorbidity patterns, and in some cases, the model predictions were far from the expected values. This highlights the difficulty in modeling total charge estimates for diseases that may interact in a multimorbidity, since they do not have a simple additive effect. This demonstrates the need to develop more robust models to ensure the healthcare system can better serve all populations. Improved modeling of underserved populations is necessary, and multimorbidity and race need to be studied more comprehensively.

### Supplementary Information

**Additional file 1:** **Supplemental Table 1.**Most Prevalent Morbidities. **Supplemental Table 2.** Study variables. **Supplemental Table 3.** Additional model estimates for the 45-64 cohort. **Supplemental Table 4.** Additional model estimates for the 65+ cohort. **Supplemental Figure 1.** Patient population. **Supplemental Figure 2.** Model residuals for the 45-64 and 65+ Cohorts. **Supplemental Figure 3.** Log and Exponential model residuals. **Supplemental Figure 4.** Mean model residuals by race.

## Data Availability

The datasets generated and/or analyzed during the current study are not publicly available as they must be licensed by Oracle Cerner (https://www.cerner.com/ap/en/solutions/data-research).

## Abbreviations

BackP: Severe back pain
BMI: Body mass index
CKD: Chronic kidney disease
ECI: Elixhauser Comorbidity Index
GERD: Gastroesophageal reflux disease
HD: Heart disease
HypT: Hypertension
ICD-10–CM: International Classification of Diseases-10th Version-Clinical Modification
LimbP: Pain in limb, hand, foot, fingers, and toes
Lip: Lipidemia
OJD: Other joint disorder
T2D: Type 2 diabetes mellitus
UHDDS: Uniform hospital discharge data set
