# Supplementary material for: Racial differences in healthcare expenditures for prevalent multimorbidity combinations in the USA: a cross-sectional study
Source: BMC Med. 2023 Oct 23;21:399. doi: 10.1186/s12916-023-03084-2 (PMC10591380; doi:10.1186/s12916-023-03084-2)
Supplement: Supplementary file 1 — Additional file 1: Supplemental Table 1.Most Prevalent Morbidities. Supplemental Table 2. Study variables. Supplemental Table 3. Additional model estimates for the 45-64 cohort. Supplemental Table 4. Additional model estimates for the 65+ cohort. Supplemental Figure 1. Patient population. Supplemental Figure 2. Model residuals for the 45-64 and 65+ Cohorts. Supplemental Figure 3. Log and Exponential model residuals. Supplemental Figure 4. Mean model residuals by race. [file 12916_2023_3084_MOESM1_ESM.docx]

**Additional File 1**

**Supplemental Table 1.** **Most Prevalent Morbidities at the 5% Threshold**

| **#** | **Diagnosis** |
| --- | --- |
| 1 | **D63:** Anemia in chronic diseases classified elsewhere |
| 2 | **D64:** Other anemias |
| 3 | **E03:** Other hypothyroidism |
| 4 | **E11:** Diabetes |
| 5 | **E55:** Vitamin D deficiency |
| 6 | **E78:** Lipidemia |
| 7 | **E86:** Volume depletion |
| 8 | **E87:** Other disorders of fluid, electrolyte, and acid-base balance |
| 9 | **F03:** Unspecified dementia |
| 10 | **F10:** Alcohol related disorders |
| 11 | **F17:** Nicotine dependence |
| 12 | **F32:** Major depressive disorder, single episode |
| 13 | **F41:** Anxiety Disorders |
| 14 | **G47:** Sleep disorders are represented |
| 15 | **G89:** Pain, not elsewhere classified |
| 16 | **I10:** Hypertension |
| 17 | **I12:** Hypertensive chronic kidney disease |
| 18 | **I25:** Heart disease |
| 19 | **I48:** Atrial fibrillation and flutter are prevalent |
| 20 | **I50:** Heart failure |
| 21 | **I65:** Occlusion and stenosis of precerebral arteries, not resulting in cerebral infarction |
| 22 | **I73:** Other peripheral vascular diseases |
| 23 | **J44:** Other chronic obstructive pulmonary disease |
| 24 | **J45:** Asthma |
| 25 | **K21:** GERD |
| 26 | **K57:** Diverticular disease of intestine |
| 27 | **K59:** Other functional intestinal disorders |
| 28 | **M17:** Osteoarthritis of knee |
| 29 | **M19:** Other and unspecified osteoarthritis |
| 30 | **M25:** Other Joint Disorders |
| 31 | **M54:** Dorsalgia |
| 32 | **M79:** Other and unspecified soft tissue disorders, not elsewhere classified |
| 33 | **M81:** Osteoporosis without current pathological fracture |
| 34 | **N17:** Acute kidney failure |
| 35 | **N18:** Chronic kidney disease (CKD) |
| 36 | **N28:** Other disorders of kidney and ureter, not elsewhere classified |
| 37 | **N39:** Other disorders of urinary system |
| 38 | **N40:** Benign prostatic hyperplasia |

**Supplemental Table 2. Study Variables.**

| **Variable** | **Notes** |
| --- | --- |
| **Comorbidity** | 38 comorbidities in Table 1 with ICD-10-CM broad categories |
| **Age** | 45+ |
| **Gender** | Male and Female |
| **Race** | African American, Asian/Pacific Islander, Biracial, Caucasian, Hispanic, and Native American |
| **BMI** | With obesity (30 ≤ BMI < 206) and without obesity (18.5 ≤ BMI < 30). 18.5 is the minimum BMI for normal weight, and 206 is the maximum BMI recorded for a human when this study was first conducted |
| **Total number of morbidities** | Added the total number of morbidities for each patient during the two years. |
| **Elixhauser Comorbidity Index (ECI)** | Assess disease burden. |
| **Total Charges** | Total charges greater than $0 were included, and the average charges of all encounters for the two years were assigned for each patient. The records in the Cerner HealthFacts database have the total charges of each patient encounter. An encounter is an interaction between a patient and healthcare provider(s) to provide healthcare service(s) or assess a patient's health status of a patient.^39^ Hospital charges are more accurate in reflecting the patient population receiving a hospital service.^40^ |
| **Payer Type** | Medicare/Medicaid/Title V, Other, and Unknown |
| **Hospital Division** | Based on the U.S. Census Bureau, nine hospital divisions were in the data set. Some studies have projected that geographical location impact cost.^41^ |
| **Length of Stay** | If the patient was hospitalized, their length of stay was calculated by subtracting the discharge date from the admission date. |
| **Number of Emergency Visits** | Adding the number of emergency room visits during the two years |
| **Number of Outpatient Visits** | Adding the number of outpatient visits during the two years |
| **Urban Status of Area** | The urban or rural status of a hospital impacts cost^42^ |
| **Teaching Status of Hospital** | Indicating the teaching status of the hospital |
| **Acute Care Status** | Hospitals providing acute care services are more profitable than other services and therefore are projected to impact cost.^43^ |

**Supplemental Table 3. Morbidity and Hospital Information** **Model Estimates for the 45-64 Cohort**

| **Morbidity (Variable)** | **Estimate** | **P-value** | **Significance** |
| --- | --- | --- | --- |
| **K57:** Diverticular disease of intestine | 0.134 | <0.001 | *** |
| **F10:** Alcohol related disorders | 0.115 | <0.001 | *** |
| **K21:** GERD | 0.096 | <0.001 | *** |
| **F17:** Nicotine dependence | 0.095 | <0.001 | *** |
| **I25:** Heart disease | 0.092 | <0.001 | *** |
| **N17:** Acute kidney failure | 0.086 | <0.001 | *** |
| **I12:** Hypertensive chronic kidney disease | 0.084 | <0.001 | *** |
| **N28:** Other disorders of kidney and ureter, not elsewhere classified | 0.083 | <0.001 | *** |
| **E86:** Volume depletion | 0.082 | <0.001 | *** |
| **D64:** Other anemias | 0.081 | <0.001 | *** |
| **M17:** Osteoarthritis of knee | 0.081 | <0.001 | *** |
| **I65:** Occlusion and stenosis of precerebral arteries, not resulting in cerebral infarction | 0.079 | <0.001 | *** |
| **E87:** Other disorders of fluid, electrolyte, and acid-base balance | 0.060 | <0.001 | *** |
| **F32:** Major depressive disorder, single episode | 0.059 | <0.001 | *** |
| **I10:** Hypertension | 0.056 | <0.001 | *** |
| **M19:** Other and unspecified osteoarthritis | 0.054 | <0.001 | *** |
| **N40:** Benign prostatic hyperplasia | 0.054 | <0.001 | *** |
| **K59:** Other functional intestinal disorders | 0.051 | <0.001 | *** |
| **E11:** Diabetes | 0.042 | <0.001 | *** |
| **G89:** Pain, not elsewhere classified | 0.041 | <0.001 | *** |
| **N39:** Other disorders of urinary system | 0.040 | <0.001 | *** |
| **J45:** Asthma | 0.038 | <0.001 | *** |
| **I48:** Atrial fibrillation and flutter are prevalent | 0.034 | <0.001 | *** |
| **G47:** Sleep disorders are represented | 0.031 | <0.001 | *** |
| **M81:** Osteoporosis without current pathological fracture | 0.029 | <0.001 | *** |
| **F03:** Unspecified dementia | 0.028 | 0.006 | ** |
| **E03:** Other hypothyroidism | 0.027 | <0.001 | *** |
| **F41:** Anxiety Disorders | 0.025 | <0.001 | *** |
| **J44:** Other chronic obstructive pulmonary disease | 0.024 | <0.001 | *** |
| **D63:** Anemia in chronic diseases classified elsewhere | 0.023 | 0.002 | ** |
| **M54:** Dorsalgia | 0.022 | <0.001 | *** |
| **E78:** Lipidemia | 0.020 | <0.001 | *** |
| **M25:** Other Joint Disorders | 0.015 | <0.001 | *** |
| **M79:** Other and unspecified soft tissue disorders, not elsewhere classified | 0.013 | <0.001 | *** |
| **I73:** Other peripheral vascular diseases | 0.011 | 0.011 | * |
| **I50:** Heart failure | -0.008 | 0.012 | * |
| **E55:** Vitamin D deficiency | -0.010 | <0.001 | *** |
| **N18:** Chronic kidney disease (CKD) | -0.054 | <0.001 | *** |

| **Hospital Information (Variable)** | **Estimate** | **P-value** | **Significance** |
| --- | --- | --- | --- |
| Urban hospital | 0.009 | <0.001 | *** |
| Acute care hospital | 0.288 | <0.001 | *** |
| Teaching hospital | 0.000 | 0.731 | - |
| Census Division 5: East South Central (A.L., KY, MS, TN) | -0.180 | 0.016 | * |
| Census Division 6: South Atlantic (D.E., DC, FL, GA, MD, NC, SC, VA, WV) | -0.185 | 0.013 | * |
| Census Division 3: West North Central (I.A., KS, MN, MO, ND, SD) | -0.260 | 0.001 | *** |
| Census Division 9: Pacific (A.K., CA, HI, OR, WA) | -0.272 | <0.001 | *** |
| Census Division 7: West South Central (A.R., LA, OK, TX) | -0.279 | <0.001 | *** |
| Census Division 4: East North Central (I.L., IN, MI, OH, WI) | -0.368 | <0.001 | *** |
| Census Division 8: Mountain (AZ, CO, ID, MT) | -0.375 | <0.001 | *** |
| Census Division 2: Middle Atlantic (N.J., NY, PA) | -0.390 | <0.001 | *** |

**Supplemental Table 4. Morbidity and Hospital Information Model Estimates for the *65+* Cohort**

| **Morbidity (Variable)** | **Estimate** | **P-value** | **Significance** |
| --- | --- | --- | --- |
| **M17:** Osteoarthritis of knee | 0.263 | <0.001 | *** |
| **K57:** Diverticular disease of intestine | 0.230 | <0.001 | *** |
| **F10:** Alcohol related disorders | 0.229 | <0.001 | *** |
| **I12:** Hypertensive chronic kidney disease | 0.219 | <0.001 | *** |
| **N17:** Acute kidney failure | 0.217 | <0.001 | *** |
| **K21:** GERD | 0.208 | <0.001 | *** |
| **I25:** Heart disease | 0.203 | <0.001 | *** |
| **I65:** Occlusion and stenosis of precerebral arteries, not resulting in cerebral infarction | 0.197 | <0.001 | *** |
| **F17:** Nicotine dependence | 0.194 | <0.001 | *** |
| **F03:** Unspecified dementia | 0.169 | <0.001 | *** |
| **I10:** Hypertension | 0.169 | <0.001 | *** |
| **E86:**Volume depletion | 0.166 | <0.001 | *** |
| **E87:** Other disorders of fluid, electrolyte, and acid-base balance | 0.160 | <0.001 | *** |
| **N40:** Benign prostatic hyperplasia | 0.155 | <0.001 | *** |
| **G89:** Pain, not elsewhere classified | 0.152 | <0.001 | *** |
| **F32:** Major depressive disorder, single episode | 0.151 | <0.001 | *** |
| **D64:** Other anemias | 0.132 | <0.001 | *** |
| **N28:** Other disorders of kidney and ureter, not elsewhere classified | 0.125 | <0.001 | *** |
| **N39:** Other disorders of urinary system | 0.111 | <0.001 | *** |
| **J45:** Asthma | 0.109 | <0.001 | *** |
| **M19:** Other and unspecified osteoarthritis | 0.107 | <0.001 | *** |
| **I48:** Atrial fibrillation and flutter are prevalent | 0.104 | <0.001 | *** |
| **J44:** Other chronic obstructive pulmonary disease | 0.102 | <0.001 | *** |
| **E78:** Lipidemia | 0.097 | <0.001 | *** |
| **D63:** Anemia in chronic diseases classified elsewhere | 0.097 | <0.001 | *** |
| **K59:** Other functional intestinal disorders | 0.097 | <0.001 | *** |
| **E11:** Diabetes | 0.096 | <0.001 | *** |
| **E03:** Other hypothyroidism | 0.092 | <0.001 | *** |
| **G47:** Sleep disorders are represented | 0.091 | <0.001 | *** |
| **F41:** Anxiety Disorders | 0.079 | <0.001 | *** |
| **M81:** Osteoporosis without current pathological fracture | 0.076 | <0.001 | *** |
| **I73:** Other peripheral vascular diseases | 0.065 | <0.001 | *** |
| **M54:** Dorsalgia | 0.056 | <0.001 | *** |
| **M25:** Other Joint Disorders | 0.041 | <0.001 | *** |
| **M79:** Other and unspecified soft tissue disorders, not elsewhere classified | 0.023 | <0.001 | *** |
| **I50:** Heart failure | 0.020 | <0.001 | *** |
| **E55:** Vitamin D deficiency | -0.074 | <0.001 | *** |
| **N18:** Chronic kidney disease (CKD) | -0.133 | <0.001 | *** |
| **Hospital Information (Variable)** | **Estimate** | **P-value** | **Significance** |
| Urban hospital | 0.023 | <0.001 | *** |
| Acute care hospital | 0.776 | <0.001 | *** |
| Teaching hospital | 0.012 | <0.001 | *** |
| Census Division 6: South Atlantic (D.E., DC, FL, GA, MD, NC, SC, VA, WV) | -0.222 | 0.182 | - |
| Census Division 9: Pacific (A.K., CA, HI, OR, WA) | -0.329 | 0.048 | * |
| Census Division 7: West South Central (A.R., LA, OK, TX) | -0.400 | 0.016 | * |
| Census Division 5: East South Central (A.L., KY, MS, TN) | -0.412 | 0.013 | * |
| Census Division 3: West North Central (I.A., KS, MN, MO, ND, SD) | -0.447 | 0.007 | ** |
| Census Division 8: Mountain (AZ, CO, ID, MT) | -0.671 | <0.001 | *** |
| Census Division 2: Middle Atlantic (N.J., NY, PA) | -0.681 | <0.001 | *** |
| Census Division 4: East North Central (I.L., IN, MI, OH, WI) | -0.686 | <0.001 | *** |

**Supplemental Figure 1. Patient Population**

**A.** Unadjusted Model Residuals for the Middle-Aged Cohort

**B.** Adjusted Model Residuals for the Middle-Aged Cohort

**C.** Unadjusted Model Residuals for the Elderly Cohort

**D.** Adjusted Model Residuals for the Elderly Cohort


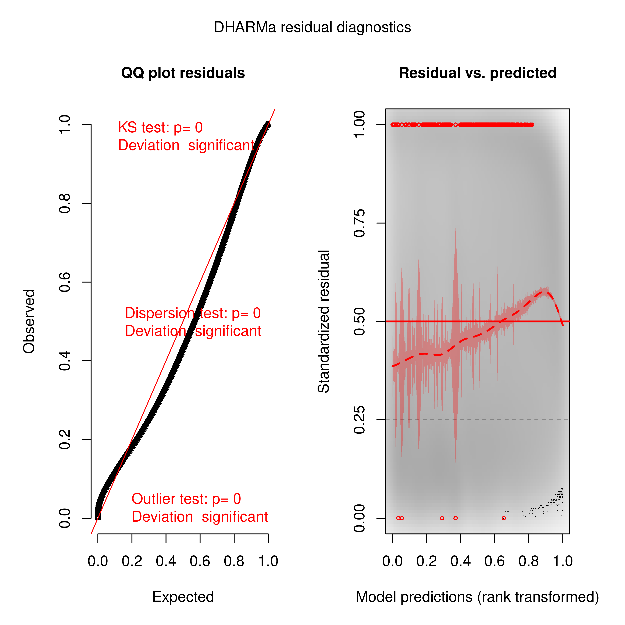

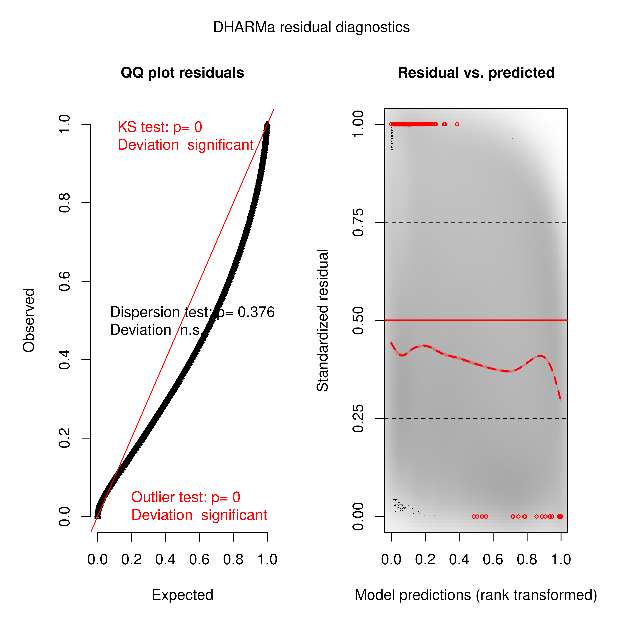

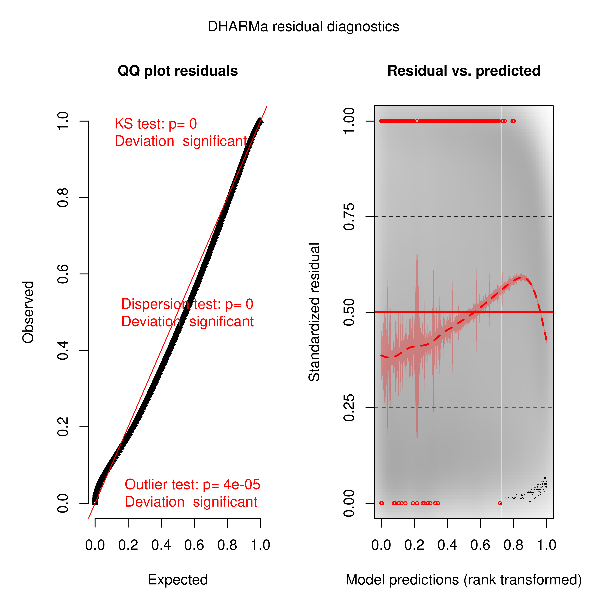

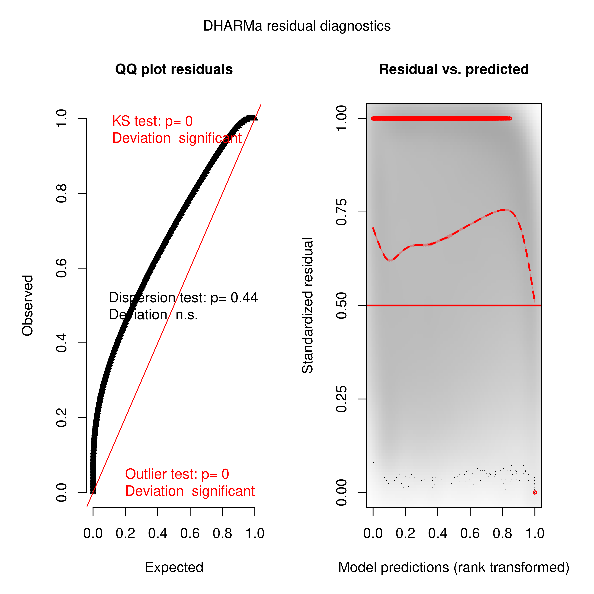


**Supplemental Figure 2. Adjusted and Unadjusted** **Model Residuals for the 45-64 and 65+ Cohorts**

**A.** Log Model Residuals for the Middle-Aged Cohort

**B.** Exponential Model Residuals for the Middle-Aged Cohort

**C.** Log Model Residuals for the Elderly Cohort

**D.** Exponential Model Residuals for the Elderly Cohort


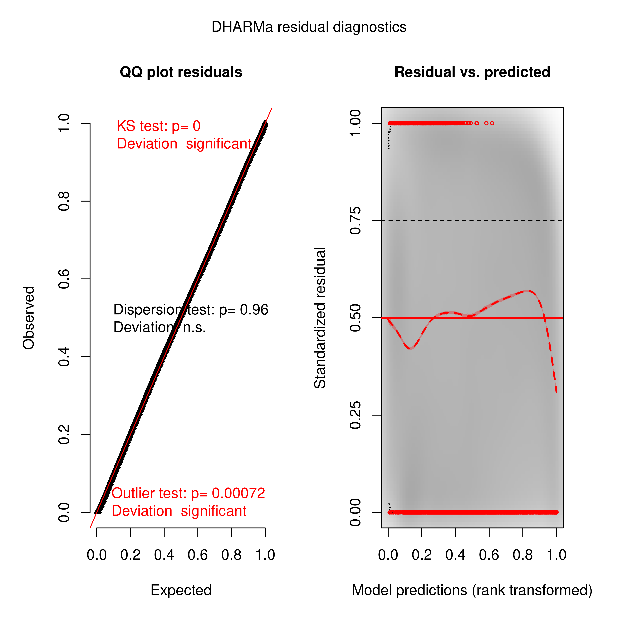

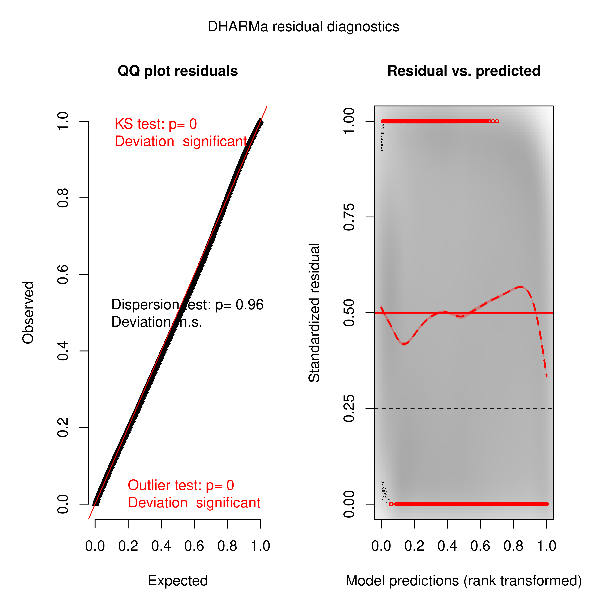

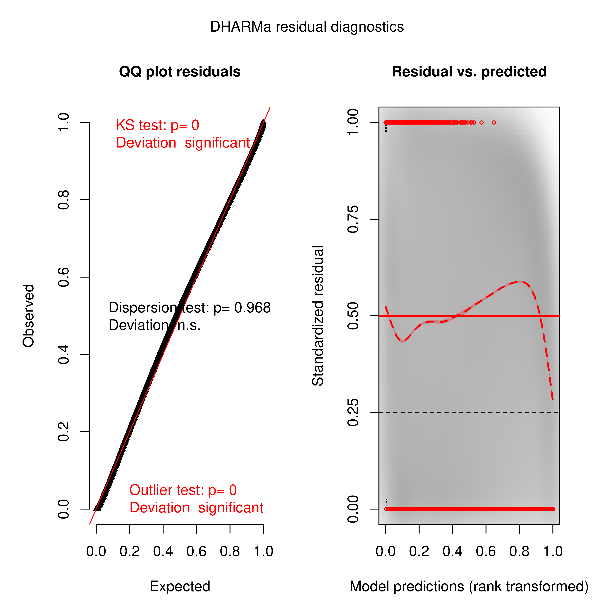

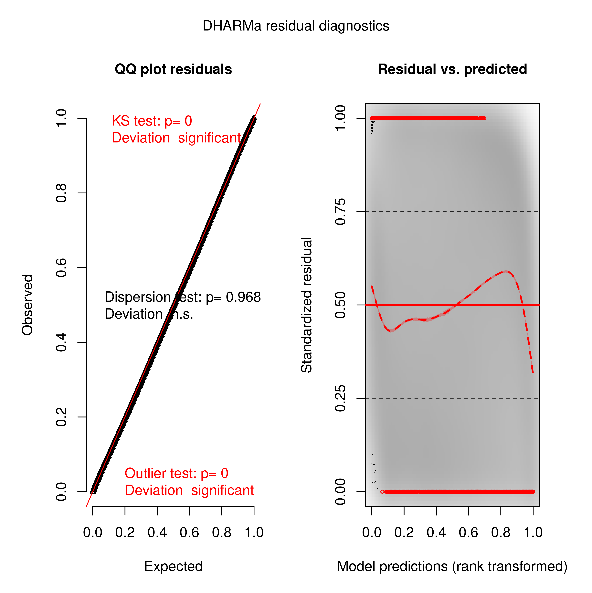


**Supplemental Figure 3. Log and Exponential Adjusted Model Residuals for the 45-64 and 65+ Cohorts**

**
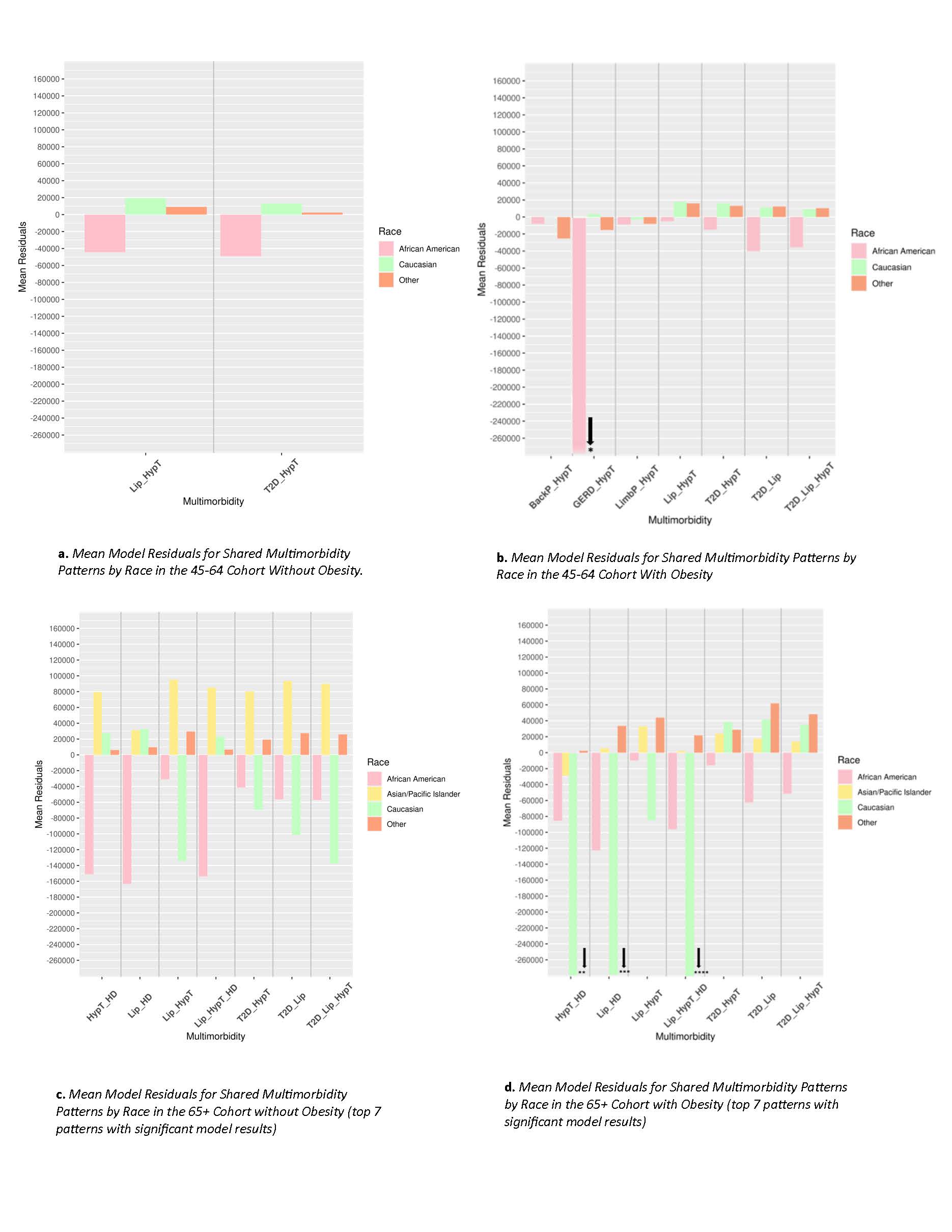
Appendix Figure 4.** **Mean Model Residuals by Race in the 65+ and 45-64 cohorts with and without obesity with Native American and Hispanic groups combined. * =** --3322569 residual value, ****** = --277376 residual value, ***= -325103 residual value, ***=-399349 residual value (these values were too large to display in the figure). **Abbreviations: BackP,** Severe back pain; **CKD,** Chronic kidney disease; **GERD,** Gastroesophageal reflux disease; **HD,** Heart disease; **HypT,** Hypertension; **Lip,** Lipidemia; **LimbP,** Pain in limb, hand, foot, fingers, and toes; **OJD,** Other joint disorder; **T2D,** Type 2 diabetes mellitus.
